# Facial Imitation Improves Emotion Recognition in Adults with Different Levels of Sub-Clinical Autistic Traits

**DOI:** 10.3390/jintelligence9010004

**Published:** 2021-01-13

**Authors:** Andrea E. Kowallik, Maike Pohl, Stefan R. Schweinberger

**Affiliations:** 1 Early Support and Counselling Center Jena, Herbert Feuchte Stiftungsverbund, 07743 Jena, Germany; 2 Social Potential in Autism Research Unit, Friedrich Schiller University, 07743 Jena, Germany; 3 Department of General Psychology and Cognitive Neuroscience, Friedrich Schiller University Jena, Am Steiger 3/Haus 1, 07743 Jena, Germany; maiapol47@gmail.com; 4 Michael Stifel Center Jena for Data-Driven and Simulation Science, Friedrich Schiller University, 07743 Jena, Germany; 5 Swiss Center for Affective Science, University of Geneva, 1202 Geneva, Switzerland

**Keywords:** emotion recognition, face, imitation, mimicry, autism, embodied cognition, facial feedback, automatic expression analysis

## Abstract

We used computer-based automatic expression analysis to investigate the impact of imitation on facial emotion recognition with a baseline-intervention-retest design. The participants: 55 young adults with varying degrees of autistic traits, completed an emotion recognition task with images of faces displaying one of six basic emotional expressions. This task was then repeated with instructions to imitate the expressions. During the experiment, a camera captured the participants’ faces for an automatic evaluation of their imitation performance. The instruction to imitate enhanced imitation performance as well as emotion recognition. Of relevance, emotion recognition improvements in the imitation block were larger in people with higher levels of autistic traits, whereas imitation enhancements were independent of autistic traits. The finding that an imitation instruction improves emotion recognition, and that imitation is a positive within-participant predictor of recognition accuracy in the imitation block supports the idea of a link between motor expression and perception in the processing of emotions, which might be mediated by the mirror neuron system. However, because there was no evidence that people with higher autistic traits differ in their imitative behavior per se, their disproportional emotion recognition benefits could have arisen from indirect effects of imitation instructions

## 1. Introduction

Humans perceive the meaning of another’s message beyond the spoken word through a range of multimodal cues, such as facial expressions, postures and gestures, and prosody. As such, emotion recognition is one important skill for successful social interactions (Koolagudi and Rao 2012). Although the expression and recognition of emotions are subject to individual and cultural variations, basic facial expressions can be recognized across cultures above chance level (cf. the meta-analysis by Elfenbein and Ambady 2002).

### 1.1. Role of Imitation

Imitation of facial expressions can already be observed in newborns (Meltzoff and Moore 1977). Beginning with the facial feedback hypothesis by Darwin ([1872] 1965), a lot of research has been conducted on the role of mimicry, or automatic imitation (Chartrand and Lakin 2013), in facial emotion recognition. It has been proposed that, through mimicking an observed emotional expression, the corresponding emotion is generated in the observer, such that the observed person’s emotional state can be inferred from the observer’s own feelings. The idea behind this fits into more recently developed theories of embodied cognition, which assume that action recognition and performing the same action share common neuronal substrates and therefore promote each other (Decety and Sommerville 2003; Foglia and Wilson 2013). Early evidence for this kind of automatic feedback process comes from Dimberg (1982), who found that looking at happy vs. angry faces resulted in differential automatic electromyographic (EMG) responses in the observers that corresponded to the activation of the observed emotional expressions. Furthermore, Wallbott (1991) found that participants could identify the emotions of an emotion recognition task solely by watching their own facial reactions from a video recording of the initial experiment. More recent EMG evidence for the role of facial muscular activity in emotion perception at the level of individual differences was provided by Künecke et al. (2014), who found a correlation between emotion-related EMG responses (of corrugator supercilii) and emotion perception ability. Specifically, participants with higher congruent EMG activity achieved better emotion recognition performance.

In a different experimental approach, Strack et al. (1988) studied the influence of artificial facial motor activation on participants’ emotionality. Participants were simultaneously watching cartoons and having a pencil in their mouth in a way that either intensified or inhibited a smiling expression. Strack et al. (1988) found that participants reported to feel more amused by the cartoons under smile-facilitating than under smile-inhibiting conditions. Oberman et al. (2007) utilized a similar design to explore the effect of artificial facial motor activation on emotion recognition. They found a selectively inferior classification of those emotions that could not be imitated, while the recognition of imitable emotions remained unaffected. Consequently, this selective influence on emotion recognition through interference with motor activity strongly suggests an important role of facial imitation in emotion recognition.

### 1.2. Autism Spectrum Conditions

Autism Spectrum Disorders or Autism Spectrum Conditions (ASC) are a group of behaviorally defined neurodevelopmental conditions that are specified by impaired reciprocal social communication and restricted repetitive patterns of behavior or activities (American Psychiatric Association 2013). Among the special communicational features, people with ASC stand out by their divergent emotional expressions. Individuals with ASC were found to have fewer facial expressions (Kasari et al. 1990) or an atypical and idiosyncratic way of expressing emotions through their faces (Brewer et al. 2016). Although also a diagnostic criterion, the evidence about emotion recognition ability in individuals with ASC is mixed (Harms et al. 2010). In a formal meta-analysis for facial emotion recognition, (Uljarevic and Hamilton 2013) found a medium overall effect size of −0.41, 95% CI [−0.646, −0.182] based on a random-effects analysis with 50 comparisons of emotion recognition between each a group with ASC and a healthy control group, corrected for the possible impact of publication bias (Duval and Tweedie’s trim-and-fill method).

Many studies on ASC focused on emotion perception, whereas less is known about the social and communicational aspects of imitation and its influence on emotion recognition. Smith and Bryson (1994) reviewed studies on the imitation ability of children with ASC. They concluded that there are imitative impairments in ASC, which can be linked to lower-level attentional and perceptual deviations. Since the discovery of “mirror neurons” (MNs; see Rizzolatti et al. 1996; Rizzolatti and Craighero 2004), the causal role of the mirror neuron system (MNS) in imitation deficits in ASC, and even in ASC itself, has been proposed (e.g., the “Broken Mirror Hypothesis” by Williams et al. 2001).

Considering imitation of emotions, McIntosh et al. (2006) found that automatic imitation of facial emotional expressions was reduced while the ability to imitate the expressions voluntarily on demand remained intact in adults with ASC. A facial feedback study on children and adolescents with ASC by Stel et al. (2008) came to the same conclusions with the additional result, that imitating emotions only elicited the corresponding emotion in the control group but not in the ASC group. (Oberman et al. 2009) obtained slightly different results, showing a delayed but otherwise normal automatic imitation in adults with ASC. In the same study, the ASC group did not differ from the control group regarding their voluntary imitation performance.

Combining imitation ability with emotion recognition, Lewis and Dunn (2017) reported an intriguing study in which they aimed at testing the thesis whether inducing voluntary imitation of facial expression promotes emotion recognition in people with high vs. low autistic traits. A promising outcome was that the instruction to mimic led to better emotion recognition results, especially in the participants with higher levels of autistic traits. An important constraint of their study was that the extent to which participants actually imitated the displayed emotions was not recorded objectively, and participants were only asked about their opinion on their imitation ability.

### 1.3. Assessment of Imitation and Scope of the Study

Many of the discussed studies relied on two major techniques to quantify emotional expressions and imitation. One of them is facial EMG, in which the electric activity of certain facial muscles is assessed. These activations can be clearly related to certain facial expressions (e.g., zygomatic region for happy and corrugator region for angry in Dimberg 1982). While the advantage is a high temporal resolution, the spatial resolution is limited by the placement of electrodes. Moreover, recording electrodes may cause irritation and direct the participants’ attention to, and interfere with, their own facial actions. The other frequently used method is the Facial Action Coding System (Ekman and Friesen 1978) that is applied by trained human raters based on video recordings of participants throughout the experiment. This offers a better spatial resolution, but the procedure is also very time consuming and needs trained raters. As a more recent approach, computer-based facial expression analysis toolkits have evolved rapidly throughout the last two decades, and provide high accuracy in laboratory settings (for general review see (Fasel and Luettin 2003); for review on recent developments and challenges see (Samadiani et al. 2019)) and correlate with EMG results, even outperforming EMG in some emotional expressions (Kulke et al. 2020). The facial behavior analysis toolkit OpenFace 2.0 (Baltrušaitis et al. 2015, 2018) provides data on a wide range of features (in particular, Action Units, but also head pose or gaze direction). These can be used for automatic facial emotion recognition (e.g., Pham et al. 2019) but also for assessing similarity between facial behaviors, which made it a valuable toolkit for our study. Computer vision approaches have also been applied to the detection of autistic traits and behaviors (see review Kowallik and Schweinberger 2019). For example, computer vision-based applications enabled checking expression production skills of basic emotions in children with ASC and providing automatic feedback (Leo et al. 2018).

While the present study follows up on the paper by Lewis and Dunn (2017), specifically their second experiment in terms of stimuli and trial structure, one of our key objectives was to quantify the degree of facial imitation in an objective and interference-free manner by using computer-based facial behavior analysis of participants´ faces during task performance. Specifically, we quantified imitation as an automatic cross-correlational comparison between the stimulus face and the participant’s facial expression, which enabled us to more directly assess the effects of the imitation instruction on both actual facial imitation and on emotion recognition performance.

## 2. Materials and Methods

### 2.1. Participants

Fifty-five undergraduate students (17 male) from various fields (27 non-social-sciences students) between the ages of 18 and 31 years (*M* = 22.96, *SD* = 3.05) contributed to the data. The data of six additional participants was excluded due to partially missing data based on technical issues. Note that the sample size was determined by power analysis (cf. Section 2.3). Participants were recruited via university e-mail-distribution lists and received either course credit or financial compensation for their participation.

### 2.2. Measures

#### 2.2.1. Autism Spectrum Quotient

The participants filled out the Autism Spectrum Quotient (AQ; Baron-Cohen et al. 2001) in the validated German Version (Freitag et al. 2007). The AQ is a self-report measurement with 50 items, screening for the degree of autistic traits. In the original publication, the average score in the control group was about 16 for men and about 15 for women. A score of 32 or more was considered a cut-off, that was reached by 80% of people with a diagnosis on the autism spectrum but only 2% of the control group.

#### 2.2.2. Stimuli 

As in the study by Lewis and Dunn (2017), images of emotional faces were taken from the “Facial Expressions of Emotion: Stimuli and Tests” (FEEST) stimuli set by Young et al. (2002). These stimuli depict six basic emotions (anger, sadness, fear, surprise, happiness, disgust) by 10 identities that are morphed with a neutral expression into four intensities (25, 50, 75, 100% emotional intensity) yielding 240 images in total. Three male and three female identities (MC02, MC05, MC06, MC07, MC08, MC10) were chosen as test identities (144 stimuli), the other ones were included in the practice trials.

### 2.3. Research Design

This study followed a baseline-intervention-re-test design. The AQ score served as a quasi-independent variable. The dependent variables were the correctness of the emotion recognition task as well as the imitation score that was computed as similarity of participants’ and stimulus’ facial expressions. A power analysis was calculated using G*Power 3 (Faul et al. 2007) for a medium effect (*d* = 0.5) with a power (1 − *β*) = 0.80 in a repeated-measures design yielding a minimum sample size of 34. For multilevel modeling, a minimum of 50 level-2 units (participants) is needed to accurately estimate SE correctly (Maas and Hox 2005).

### 2.4. Procedure

Participants were placed individually at a computer, about 60 cm away from the screen. A regular off-the-shelf webcam (logitech C270 HD webcam or logitech C920 HD PRO webcam) was placed on top of the screen (resolution 480 × 640 px, about 10 fps) directed at the participant‘s face and upper body.

Participants gave written consent and filled out the AQ. Subsequently, the emotion recognition task was carried out in a Python-based experiment using Psychopy2 V1.82 (Peirce 2007) with OpenCV2 (Bradski 2000) aligning behavioral task and image recording. The experiment consisted of a practice block, a baseline block, an instruction to mimic, and an imitation block. All instructions were given on the screen. The practice block introduced the key assignment (one key per emotional category) covering images of four identities each expressing all six emotions in a randomized order; images shown during practice were not included in the subsequent experimental blocks. Practice trials consisted of a 500 ms ISI, a 500 ms fixation cross, followed by the stimulus image for 1500 ms and then a blank screen with the response options until a key was pressed. After each practice trial, feedback on the key pressed and the correctness of answer was given. The baseline block opened with the instruction to “carefully watch the whole face presented” and included 144 trials (images of 6 emotions × 6 identities × 4 intensities) in a randomized order. Trials in the experimental blocks (baseline und imitation) were constructed similarly to the practice trials but did not include feedback. Breaks of individual length were included after every 48 trials in the two experimental blocks. Camera recording started with stimulus onset and ended with the key-pressing response to the emotion recognition task, thereby defining our visual observation window. Before entering the intervention block, participants were instructed to “mimic the facial expression before each response” to the emotion recognition task. The imitation block repeated the same 144 images in a newly randomized order with the same trial structure as in the baseline block. Key presses, reaction times, and camera images were logged for every trial.

### 2.5. Data Preprocessing and Analysis

All test stimuli (FEEST, *N* = 144) as well as every image frame obtained from the camera during the experiment (*N* = 185,898) were analyzed with the OpenFace 2.0 algorithm (Baltrušaitis et al. 2015, 2018). Frames that did not reach a confidence of 0.80 for the facial landmark detection were excluded from further analysis (*N* = 4837, or 2.6%). Then, scores for Facial Action Units (AUs; selection displayed in Table 1) were generated with an intensity between zero and five. We defined imitation as the uniform activity or inactivity of each available AU. Using SciPy (Virtanen et al. 2020) and especially the pandas package (McKinney 2010), pairwise correlations between the stimulus’ facial expression (in AUs) and each frame of the participant’s facial expression (in AUs) were computed for each trial. The highest resulting cross-correlation in each trial was taken as its imitation score. Related SPSS syntax and Python code as well as processed and anonymized data of the participants can be retrieved at Supplementary Materials (https://osf.io/gmjh6/).

The final analyses had two main objectives. First, we wanted to assess the between-subjects effect of the AQ score on the facial emotion recognition (FER) performance and on the imitation performance, as well as their changes following the imitation instruction. Second, we were interested in the within-subjects effect of imitation on FER performance, using imitation as a predictor for correct responses in each trial.

For the statistical analysis of the between-subject effects, block-wise means for FER and imitation scores were computed to calculate repeated measures analyses of covariance (ANCOVAs). Cohen’s *d* was further calculated to estimate the effect sizes of the imitation-intervention on our outcomes.

To assess within-subjects’ effects, a multilevel logarithmic regression model was chosen for its ability to take the dependency of data into account, e.g., nest responses within participants. In our experiment, the trials were nested in participants, and block was the repeated measure (*x*1*_ij_*). Stimuli were treated as level-1 and participants as level-2, both also added as random effects. The participant-centered imitation score (*x*2*_ij_* level-1 effect; for the within-participant variation was the main focus of this analysis) and the grand-mean centered AQ (***X_j_*** level-2 effect) were added as fixed effects. A predicted level-1 interaction of imitation × block as well as two cross-level interactions, AQ × imitation and AQ × block should be added. This complex model (Equation (1)) should be tested against more restricted ones. All final statistical analyses were obtained with SPSS 25.0 (IBM Corporation 2017), except for comparing model fits which were accomplished following the guidelines by Sommet and Morselli (2017).

(1) $$\begin{array}{c} Logit\;\left(\frac{P\left(Y_{i}=1\right)}{1-\;P\left(Y_{i}=1\right)}\right)\;=\;B_{00}+\left(B_{10}+\;u_{1j}\right)\times x1_{ij}+B_{20}\times x2_{ij}+\;B_{01}\times X_{j}\;+B_{11}\times x1_{ij}\times X_{j}+B_{21}\times x2_{ij}\times X_{j}+u_{0j}+u_{i0}{}_{}\\ i\;=\;\mathrm{stimulus}\\ j\;=\;\mathrm{participant} \end{array}$$

## 3. Results

### 3.1. Descriptives

#### 3.1.1. Autistic Traits

AQ scores ranged from 3 to 32 (*M* = 15.69, *SD* = 6.62), indicating substantial variability of autistic traits in this neurotypical sample. This distribution is also aligning very well with the AQ scores in the validation study by Baron-Cohen et al. (2001). 

#### 3.1.2. Baseline Block

**Figure 1:**
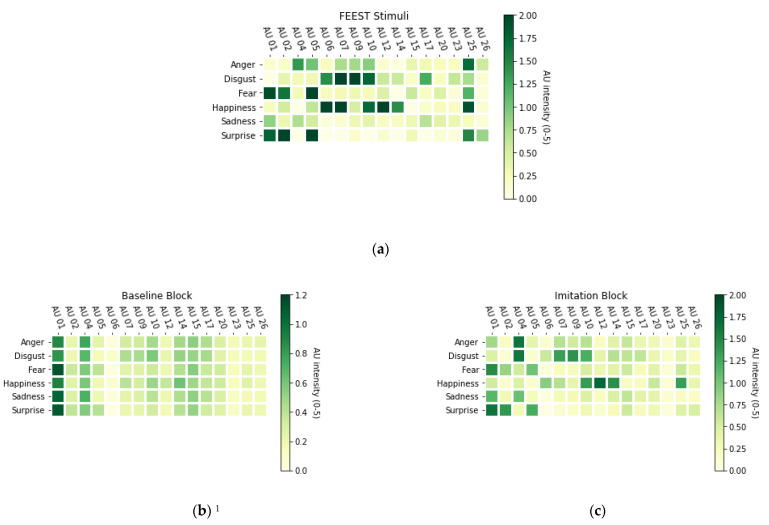
The distribution of the raw, grand-averaged AU results for (**a**) the 100% emotion FEEST stimuli and (**b**) participant’s emotional expressions while observing these stimuli in the baseline block and (**c**) the imitation block. ^1^ Note the different scaling in panel (**b**) to support visibility of subtle effects.

The mean baseline accuracy of the FER, choosing the right one out of six emotions, was *M* = 0.663, *SD* = 0.067, which is substantially above the chance rate of 1/6, or 0.167. The mean baseline cross-correlation score for imitation was *M* = 0.108, *SD* = 0.09, representing an overall small positive cross-correlation. Figure 1b shows the distribution of the averaged raw participant’s expressions (in AUs). It is apparent that AU 01 (Inner Brow Raiser) and AU 04 (Brow Lowerer) are most activated across all emotions. Albeit very small, the mean AU activations also reflect some patterns that can be expected based on the emotional stimuli they are supposed to imitate, see Figure 1a.

#### 3.1.3. Intervention Effects

The imitation intervention increased correctly recognized emotions in the imitation block (mean proportion change 0.012, 95% CI [0.00017; 0.024], *t*(54) = 2.033, *p* = 0.047). Of all 55 participants, 31 (56.4%) were able to increase their recognition performance in the imitation block, 6 (10.9%) had equivalent performance and 18 (32.7%) had a lower performance in the imitation block. For the imitation performance, almost all participants (54, or 98.2%) were able to increase their performance in the imitation block. As expected, there was a prominent increase (as seen in an increase in the mean cross-correlation coefficients of 0.171, 95% CI [0.148; 0.194], *t*(54) = 14.680, *p* < 0.001) in imitation performance in the imitation block. Figure 1c indicates that the participants’ expressions now reflect the expected AU-patterns to a much greater extent. For intervention effects, see Table 2.

### 3.2. Between-Subjects Effects

#### 3.2.1. Repeated Measures ANCOVAs

Due to the heterogeneity of covariances, two separate repeated measures ANCOVAs, one for the recognition accuracy and one for imitation performance, were computed. The continuous AQ score was treated as a covariate. A repeated measures ANCOVA for recognition accuracy did not show a statistically significant main effect of block, *F*(1, 53) = 2.428, *p* = 0.125, *η*_p_^2^ = 0.044. Thus, although there was a mean change in recognition accuracy from baseline to imitation, the intervention effect on recognition accuracy was not significant when AQ was considered as a covariate. Instead, there was a significant moderation of the block effect by AQ score *F*(1, 53) = 6.140, *p* < 0.05, *η*_p_^2^ = 0.104. This effect reflects that participants with higher AQ scores exhibited larger emotion recognition improvements from the baseline to the imitation block, compared to participants with lower AQ scores. For facial imitation scores, on the other hand, the repeated measure ANCOVA showed a significant difference between the blocks (*F*[1, 53] = 33.364, *p* < 0.001, *η*_p_^2^ = 0.386), with higher imitation scores in the imitation block as expected, but no moderation by AQ score, *F*(1, 53)= 0.289, *p* = 0.593, *η*_p_^2^ = 0.005. 

#### 3.2.2. Regression with AQ

**Figure 2:**
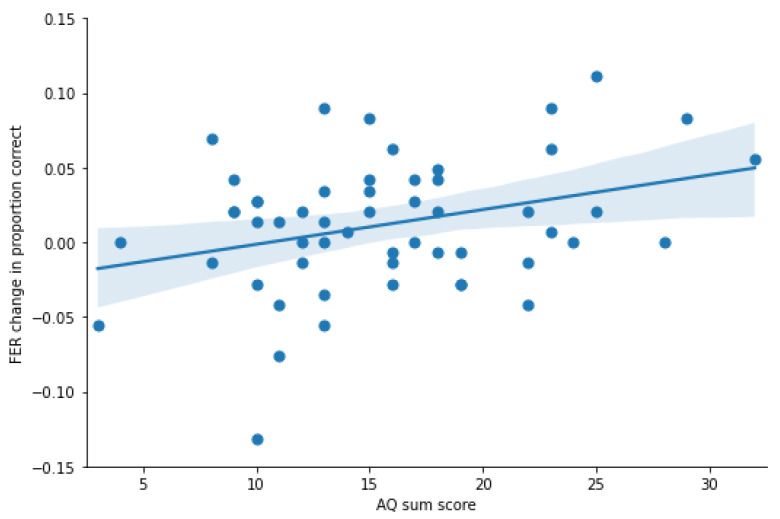
Change in FER accuracy (proportion correct) from baseline to intervention. The FER change was moderated by AQ score. Fitted linear regression with 95% CI.

As the separate repeated ANCOVAs did not investigate interactions of change in imitation and recognition accuracy, a regression of imitation change and AQ on change in emotion recognition performance was conducted, resulting in *R*^2^ = 0.105, *F*(2, 52) = 3.064, *p* = 0.055. Excluding the imitation change, which was a non-significant predictor, again, only the AQ score was predicting change in recognition performance, resulting in *R*^2^ = 0.104, *F*(1, 53) = 6.140, *p* < 0.05. Overall, this analysis did not reveal an effect of FER change as a result of imitation change per se, but the FER change in the imitation block was moderated by AQ score (Figure 2).

### 3.3. Within-Subjects Effects

As the next step, trial-wise comparisons were conducted to assess the degree to which imitation was linked to the accuracy of emotion recognition within trials. For this purpose, a multilevel logistic regression was conducted with *N* = 15,734 trials (level 1) by *K* = 55 participants (level 2). Block was again treated as a repeated measure. A participant-centered imitation score and block (level-1 effects), as well as a grand-mean-centered AQ (level-2 effect), were added as fixed effects, whereas stimuli and participants were treated as random effects. Additionally, imitation × block was added as level-1-interaction while AQ × imitation and AQ × block were added as cross-level interactions. Although AQ was not the main interest here, the related fixed effects were included to reduce unexplained variance. Model comparisons are displayed in Table 3. 

The basic model (Model 1) resembles the general tendency for more correct than incorrect emotion recognition answers (intercept). The random variance components of stimuli and participants reveal a significant variance between units. In model 2, the fixed effects block, imitation, and AQ, as well as the imitation × block level-1 interaction were added. While imitation (in a given trial) did not influence recognition accuracy overall, higher imitation did predict higher recognition accuracy in the imitation block. Interestingly, there was no additional effect of block, suggesting that emotion recognition performance in the baseline and imitation block differed mainly because of different degrees of imitation. Model 3 added the cross-level effects of AQ × imitation and AQ × block. Changes in the model were that the general negative effect of AQ on recognition accuracy became a trend, which was qualified by the AQ × block interaction. In line with the results from the between-subjects analyses reported above, this interaction indicates that people with a higher AQ had a greater recognition improvement in the imitation block than those with lower AQ. Further, the AQ × imitation interaction was non-significant. Note that this was also not to be expected, because we decided to use participant-centered rather than grand-mean-centered imitation scores (which focus on within-participant effects while normalizing for individual differences in the overall degree of imitation). The main finding regarding within-participant effects was that imitation improved recognition accuracy in the imitation block.

## 4. Discussion

### 4.1. Replication Aspects

In this study, we found that both emotion recognition performance and imitation performance could be improved by the simple instruction to imitate. On the one hand, our results therefore partly replicate the findings by Lewis and Dunn (2017), in the sense that the instruction to imitate increased emotion recognition performance, and that imitation-related improvements were larger in people with a higher AQ. On the other hand, Lewis and Dunn (2017) offered the interpretation that this disproportional benefit was because people with higher AQ scores are less likely to spontaneously imitate without instruction, such that they would show larger emotion recognition benefits from voluntary imitation via embodied cognition. The present results on actual imitation performance are particularly relevant to evaluate this interpretation because Lewis and Dunn (2017) had not actually measured imitation in their experiments. In the present study, we found that whereas the improvement in emotion recognition from the baseline to the imitation block was positively associated with the AQ score, the degree of enhancement of actual imitation was not. These findings on imitation behavior are therefore difficult to reconcile with the above interpretation by Lewis and Dunn (2017). 

On another note, the effect of the imitation instruction on emotion recognition accuracy, although statistically significant, was small. There also was a relatively large proportion (32.7%) of participants who showed a negative FER change under imitation instructions, although note that this proportion is not categorically different from the one found in the study by Lewis and Dunn (2017), in which the same was true for 23.3% of their intervention group with *N* = 30 (Exp. 2, comparable to ours). In our view, such findings are not unexpected for combinations of relatively small effect sizes and limitations in measurement precision but may call for more powerful designs in the future. Given that Lewis and Dunn (2017) did not find an effect from mere repetition of stimuli in their no-intervention group, we had opted against a no-intervention group in the present study design. This decision appears to be further legitimated by the finding that the factor block was not significant per se when the effect of imitation was considered (see Section 3.3). We would like to note that it remains a possibility that facial emotion recognition in people with a higher AQ could have disproportionately benefited from repeated exposures to the stimuli, rather than from imitation instructions per se. In our view, this alternative interpretation is not very likely, particularly when considering other findings which indicate that neuronal repetition effects are unrelated to autistic traits (Ewbank et al. 2016). If anything, repetition effects are even reduced in people with a diagnosis of ASC, and particularly so for face stimuli (Ewbank et al. 2017).

As a limitation, note that we did not collect information about any diagnoses in the unselected sample of university students that participated in the present study. Although we think that it is unlikely that this affected the present results, we therefore cannot exclude the possibility that a few individual participants may have been affected by anxiety, depression or other conditions that could have influenced emotion recognition. 

### 4.2. New and Technical Aspects

In the present study, we developed a new method to quantify facial imitation that is independent of emotional labels but relies exclusively on the shared expression of participant and stimulus face, in terms of automatically classified activation patterns of facial action units, using the OpenFace toolkit (Baltrušaitis et al. 2015). As the automatic expression analysis was not reported as unpleasant by any participant, it seems to be an objective and irritation-free tool to assess facial expressive behavior, in both the general population and probably even in people with autism. Indicating a degree of spontaneous imitation, we could demonstrate that the imitation score as cross-correlation of AUs between stimuli and participant’s face was significantly greater than zero (see Section 3.1.2), even when people were not actively instructed to imitate (in the baseline block). Note also that the present imitation score for the imitation block was much larger than the one during baseline, and that a common effect size estimator (Cohen’s *d* = 1.963) indicated that this is a very large effect. Further, it could be noted that although the imitation score during the imitation block may appear moderate in absolute terms, a cross-correlation of 1 would require all 16 Action Units to be perfectly aligned between stimulus’ and participant’s facial expressions. The present short stimulus presentation (1.5 s) and the concurrent task demands (emotion recognition) may have been limitations to obtaining higher imitation scores. It should also be noted that AU 01 and AU 04 were relatively active throughout all baseline trials, probably reflecting the mental state of concentration. In fact, AU 04 (the corrugator), was characterized as the “muscle of concentration” already by Darwin (Ekman 2003). 

It is also an interesting finding that our neurotypical sample was in fact able to substantially increase their imitation performance for an extended period of time on the basis of a simple instruction. Our detailed examination of the individual trials showed that a higher imitation score was only associated with the recognition accuracy in the imitation block. In the baseline block, where imitation scores were much lower overall, this effect could not be demonstrated. This could be due to technical limitations, such as reduced sensitivity for subtle facial changes. Alternatively, it might also be due to the fact that the static black and white stimuli from the FEEST were not particularly strong triggers for spontaneous imitation, that participants were focused on the task, or both. In future imitation studies, it might be rewarding to use dynamic emotional faces and task contexts that are more related to real-life interactions. 

### 4.3. Future Perspectives

The present study did not provide evidence for a link between autistic traits and either spontaneous or voluntary imitation. Although this contradicts the (untested) hypothesis by Lewis and Dunn (2017), our results seem more in line with findings of preserved voluntary imitation of facial expression in individuals with ASC (McIntosh et al. 2006; Stel et al. 2008). When considering potential technical limitations to quantify subtle effects of spontaneous imitation (see Section 4.3) in combination with the evidence for reduced spontaneous imitation in ASC which is somewhat controversial (McIntosh et al. 2006; Oberman et al. 2009), the lack of association between autistic traits and imitation may not be entirely unexpected. Nevertheless, we believe that it may be promising to pursue the present research with a refined and extended setup (e.g., with stimuli that promote larger degrees of imitation, with participants with a clinical diagnosis of ASC, and with more refined automated facial expression analysis methods). 

Even though imitation instructions did not promote disproportional enhancements in facial imitation in people with higher AQ, people with higher AQ exhibited larger benefits of imitation instructions for emotion recognition performance. While this finding seems challenging to explain, we tentatively suggest that the benefits to emotion recognition of people with high AQ are not directly linked to more facial imitation per se, but rather to a different way of processing that is promoted by imitation instructions. For instance, such instructions could attenuate or eliminate a reduction in social attention or mentalizing in people with high AQ. Recent research has shown differences in brain processing of the very same facial expressions depending on whether participants engage in emotion recognition or mentalizing tasks (Kang et al. 2018), and also that people with high AQ may have reduced spontaneous mentalizing (Nijhof et al. 2017). Albeit plausible, we wish to make explicit that this is a speculation that was not based on a priori hypotheses, and thus would need to be tested in a more systematic manner. At the same time, it seems clear that systematic future empirical research will benefit from a coordinated development of both theories about psychological constructs and their operationalization/measurement (Olderbak and Wilhelm 2020).

Although the face is a prominent vehicle for emotional communication, emotions are also powerfully transmitted via the human voice or via body motion (Castellano et al. 2008). Much of the available evidence suggests a tight correspondence between impairments in facial and vocal emotion recognition (Gray and Tickle-Degnen 2010; Philip et al. 2010; for a recent review, see Young et al. 2020). Although comparatively little research exists on imitation of vocal characteristics during auditory communication, it would be an interesting question for future research whether instructions to imitate voices or bodily motions, can potentially enhance emotion recognition in the respective sensory domains as well. 

Regarding the potential effects of participant age and sex, we note that the young adult (18–31) age range of the present sample coincides with a performance peak of emotion recognition abilities during adulthood (Olderbak et al. 2019). As a limitation, our sample was predominantly female, such that we did not analyze sex differences. While female participants consistently outperform males in facial emotion recognition, there is a relative lack of research on sex differences in facial imitation (but see Sonnby-Borgström et al. 2008).

As a result of the delayed response mode in the present experimental paradigm, we also have no information about the point in time at which participants recognized the facial emotion. It is reasonable to assume that a correctly recognized emotion is substantially easier to imitate. In that sense, both the time course and correctness of overt emotion recognition could be a moderator for imitation behavior. This issue could be addressed in future studies that record immediate behavioral responses, real-time indicators of neuronal processing such as EEG or MEG, or both (Schirmer and Adolphs 2017).

## Figures and Tables

**Table 1:** Action Units (AUs) used in this study.

AU Number	Facial Action Code Name ^1^	Muscular Basis ^1^	Associated Emotional Expressions ^2^
01	Inner Brow Raiser	Frontalis, Pars medialis	Fear, Sadness, Surprise
02	Outer Brow Raiser	Frontalis, Pars lateralis	Surprise
04	Brow Lowerer	Depressor, Glabelle, Depressor supercilii, Corrugator	Fear, Sadness, Disgust; Anger
05	Upper Lid Raiser	Levator palpebrae superioris	Surprise, Fear
06	Cheek Raiser	Orbicularis oculi, Pars orbitalis	Happiness
07	Lid Tightener	Orbicularis oculi, pars palpebralis	Anger, Disgust
09	Nose Wrinkler	Levator labii superioris alaquae nasi	Disgust
10	Upper Lip Raiser	Levator labii superioris, Caput infraorbitalis	Happiness
12	Lip Corner Puller	Zygomaticus major	Happiness
14	Dimpler	Buccinator	
15	Lip Corner Depressor	Depressor anguli oris (Triangularis)	Sadness
17	Chin Raiser	Mentalis	Anger, Sadness, Disgust
20	Lip Stretcher	Risorius with platysma	Fear
23	Lip Tightener	Orbicularis oris	Anger
25	Lips Part	Depressor Labii inferioris, or relaxation of Mentalis, or Orbicularis Oris	
26	Jaw Drop	Masetter; relaxed Temporalis and internal Pterygoid	Happiness

^1^ The information was gathered from Cohn et al. (2007). ^2^ Top 4 discriminative AUs for each basic emotion as derived from Velusamy et al. (2011).

**Table 2:** Descriptive statistics and change estimator.

	Baseline Block		Imitation Block		Change		
	*M*	*SD*	*M*	*SD*	*M*	Cohen’s *d* ^1^	95% CI ^2^
FER accuracy ^3^	0.663	0.067	0.675	0.066	0.012	0.177	[−0.158; 0.512]
Imitation ^4^	0.108	0.09	0.279	0.09	0.171	1.963	[1.535; 2.337]

^1^ Cohen’s *d* was calculated on the mean change given the averaged SDs of baseline and imitation block. ^2^ Note that the 95% CIs refer to the estimator Cohen’s *d* and not the mean change. ³ Mean proportion correct. ^4^ Mean of cross-correlation coefficients.

**Table 3:** Model comparison of multilevel logistic regressions on changes in recognition accuracy.

Effect	Model 1	Model 2	Model 3
	Fixed effects		
Intercept	1.10 *** (0.16)	1.04 *** (0.17)	1.04 *** (0.16)
Level-1			
Block (=1)		0.07 (0.04)	0.06 (0.04)
Imitation		−0.07 (0.11)	−0.05 (0.11)
Imitation * Block (=1) ^2^		0.40 ** (0.15)	0.40 ** (0.15)
Level-2			
AQ		−0.01(0.01)	−0.02 ^†^ (0.01)
Cross-level			
AQ * imitation			−0.01 (0.01)
AQ * Block (=1) ^2^			0.01 ** (0.01)
	Random Effects		
Variance component			
Level-1 (stimulus)	3.318 *** (0.425)	3.271 *** (0.419)	3.232 *** (0.419)
Level-2 (participant)	0.176 *** (0.038)	0.177 *** (0.039)	0.177 *** (0.039)
	Goodness of fit		
Deviance ^1^	78,927.384	77,992.939	77,999.512
Δ*χ* ^2^		934.445	927.863
Δ*df*		4	6
*p*		0.000	0.000

Note. For each model, coefficient estimates are given, SE are presented in brackets. ^†^ *p* < 0.10 * *p* < 0.05, ** *p* < 0.01, *** *p* < 0.001. ^1^ -2 Log-Likelihood, ^2^ The coefficients for Block = 0 have been set to zero because of redundancy.

## Supplementary Materials

The following materials are available online at https://osf.io/gmjh6/, Anonymized Data: participant-mean data, trial-wise data; SPSS syntax, python code for cross-correlation.
